# Peak oxygen uptake in older adults with heart failure: a systematic review and meta-analysis

**DOI:** 10.1007/s11357-025-01795-3

**Published:** 2025-07-28

**Authors:** Veronika Schmid, Sarah Paterson, Christopher Weinkauf, Jing Wang, Corey R. Tomczak, David Niederseer, Jan Vontobel, Daniel E. Forman, Martin Halle, Michael D. Nelson, Stephen J. Foulkes, Mark J. Haykowsky

**Affiliations:** 1 Department of Preventive Sports Medicine and Sports Cardiology, School of Medicine and Health, TUM University Hospital, Technical University Munich (TUM), Am Olympiapark 11, 80809 Munich, Bavaria, Germany; 2 Integrated Cardiovascular Exercise Physiology and Rehabilitation Lab, Faculty of Nursing, College of Health Science, University of Alberta, Edmonton, AB Canada; 3 Division of Public Health, School of Medicine, University of Utah, Salt Lake City, Utah, USA; 4 College of Kinesiology, University of Saskatchewan, Saskatoon, SK Canada; 5 Hochgebirgsklinik Davos, Medicine Campus Davos, Davos, Switzerland; 6 Christine Kuehne Center for Allergy Research and Education (CK-CARE), Medicine Campus Davos, Davos, Switzerland; 7 Department of Cardiology, Center of Translational and Experimental Cardiology (CTEC), University Heart Center Zurich, University Hospital Zurich, University of Zurich, Zurich, Switzerland; 8 Education, and Clinical Center (GRECC), Geriatric Research, VA Pittsburgh Healthcare System, Pittsburgh, PA USA; 9 Department of Medicine, Divisions of Geriatrics and Cardiology, University of Pittsburgh, Pittsburgh, PA USA; 10 Department of Kinesiology, University at Texas, Arlington, Arlington Texas, USA; 11 Heart, Exercise and Research Trials Lab, St Vincent’s Institute of Medical Research, Fitzroy, VIC Australia

**Keywords:** Aging, Frailty, Oxygen consumption, Exercise capacity

## Abstract

Reduced peak oxygen uptake (V̇O₂peak) is a hallmark of heart failure (HF) and aging, but studies on V̇O₂peak in older HF patients are limited. We compared V̇O₂peak in older HF patients versus controls and investigated differences across the age continuum: young-old (YO, 60–69 years), middle-old (MO, 70–79 years), and oldest-old (OO, ≥ 80 years). PubMed was searched (1967 May 2024) for studies meeting the following inclusion criteria: (1) HF patients with mean age ≥ 65 years; (2) V̇O₂peak measured via maximal cardiopulmonary exercise testing, and (3) control group for aim 1. For aim 2, studies had to compare V̇O₂peak within the age-subgroups. Random-effects meta-analysis was conducted, comparing V̇O₂peak (primary aim, and Fick determinants when reported) between HF and controls (aim 1), and V̇O₂peak between older age-subgroups (aim 2). Results are presented as weighted mean differences (WMD) with 95% confidence intervals. From 2788 screened articles, 38 studies were included. For aim 1, 30 studies (HF: *n* = 1093, mean age 70 years, 46% female; controls: *n* = 942, mean age 69 years, 52% female) demonstrated a significantly lower V̇O₂peak in HF (WMD − 8.8 mL/kg/min, [95% CI] − 10.3 to − 7.3 mL/kg/min). In the subset of patients where Fick determinants were measured, peak cardiac output and heart rate were lower in HF versus controls. For aim 2, eight studies showed progressively lower V̇O₂peak with age: MO vs. YO (− 1.5 mL/kg/min) and OO vs. MO (− 1.2 mL/kg/min). Older HF patients exhibit significant V̇O₂peak reductions compared to controls, with progressively lower V̇O₂peak values observed across the older age continuum.

**Graphical abstract:**

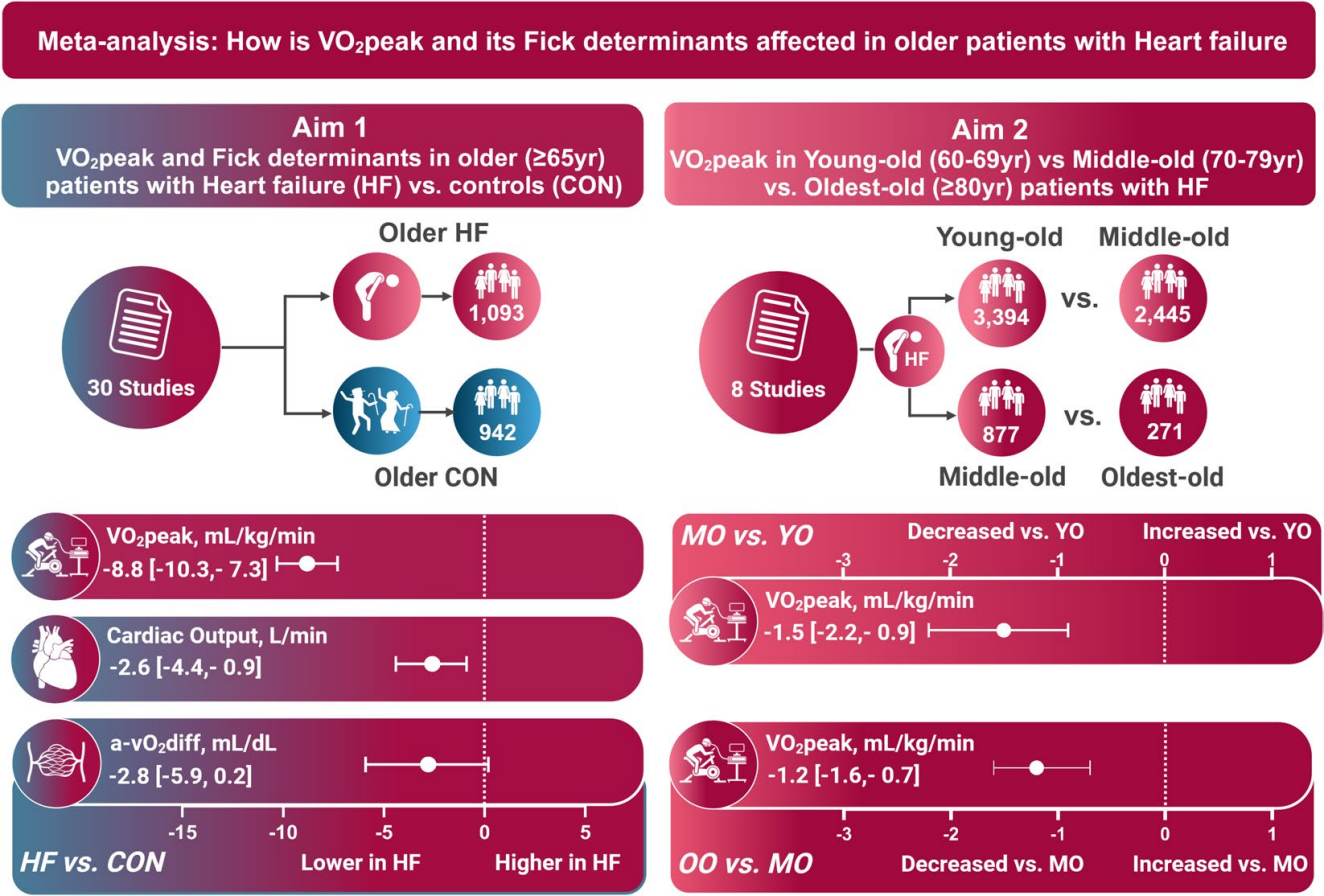

**Supplementary Information:**

The online version contains supplementary material available at 10.1007/s11357-025-01795-3.

## Introduction

Heart failure (HF), often considered a cardiogeriatric syndrome due to its rising incidence with age, affects 1–3% of the general adult population, rising to 8–10% among those over 80 years old [1]. This increase is particularly driven by the growing prevalence of HF with preserved ejection fraction (HFpEF) among older adults. Aging is also associated with a pronounced increase in the risk of frailty and disability, partly due to the age-related reduction in the reserve of multiple organ systems [1]. However, the risk and development of frailty can also be markedly accelerated by chronic diseases, such as HF, that further reduce physiological reserve.

With advancing age, peak oxygen uptake (V̇O₂peak) declines, and by the time individuals reach the seventh or eighth decade of life, this reduction, especially when coupled with sedentary deconditioning, makes activities of daily living require near-maximal to maximal effort. Consequently, this significantly reduces functional capacity and worsening quality of life [2, 3]. Marked impairment in V̇O₂peak is also a hallmark feature of HF, regardless of whether this is HFpEF or HF with reduced ejection fraction (HFrEF). The diminished V̇O₂peak in HF has been attributed to impaired reserve in cardiac and peripheral “non-cardiac systems,” many of which can also be negatively impacted by aging itself [4]. However, the degree to which reductions in V̇O₂peak and physiologic reserve associated with HF and aging interrelate has received less attention. Indeed, despite a high prevalence and poor outcomes, older individuals (≥ 65 years) with HF are underrepresented in clinical trials, leaving the specific effects of aging on V̇O₂peak, and its progression across age groups largely unexplored [5]. This is particularly important to understand, as the pronounced increase in incidence and prevalence of HF in older age and the potential additive or interactive effects of age and HF on V̇O₂peak could place older patients with HF at substantial risk of frailty and disability.

The primary aim of this systematic review and meta-analysis was to assess V̇O₂peak in older (mean age ≥ 65 years) individuals with HF compared to non-heart failure controls (CON), and to examine the Fick determinates of V̇O₂peak (when reported). A secondary aim was to compare V̇O₂peak in HF patients across the older age spectrum, including young old (YO, 60–69 years), middle old (MO, 70–79 years), and oldest old (OO, ≥ 80 years) [6].

## Methods

### Search strategy and selection criteria

In this systematic review and meta-analysis, we assessed studies that examined differences in V̇O₂peak between older adults with HF and CON (aim 1), as well as between HF patients across the older age continuum (YO vs. MO and MO vs. OO, aim 2). A search of the PubMed database was performed for English-language articles published between 1967 and May 2024. The search strategy employed the following terms: (“heart failure” OR “heart failure pathophysiology” OR “heart failure diagnosis”) AND (“exercise tolerance” OR “oxygen consumption” OR “peak VO_2_” OR “cardiorespiratory fitness”) AND (“elderly” OR ‘old’ OR “aged”). Additionally, a manual search of references from studies included in this review was conducted via the “Cited by” function on Google Scholar. The literature searches were restricted to English language articles.

Studies found through the database search were exported in full text and imported into the Covidence review management software (Melbourne, Australia). Each study was initially screened by title and abstract, followed by a full-text review if it met the specified inclusion criteria. Subsequently, data from all included studies were extracted for the following variables (number of patients, age, sex, HF subtype, and outcome measures) and were independently evaluated by two investigators (CW, SP). In cases where studies contained duplicate data, the one with the most pertinent information was selected to prevent overlapping populations. As this study used data solely from previously published research without any personally identifiable information, ethical approval was not required.

To be included, studies had to fulfil the following criteria: (1) participants with HF (HF with reduced or preserved ejection fraction [HFrEF, HFpEF]) with a mean age of 65 years or older; and (2) VO₂peak assessed through maximal cardiopulmonary testing. For aim 1, an additional requirement was a control group without HF. For aim 2, studies needed to compare V̇O₂peak values in HF patients across the older age continuum as previously defined (see aim section) [6]. Exclusion criteria were as follows: (1) lack of peak exercise data; (2) non-original or duplicate data; (3) studies on non-human subjects (e.g., animal models); or (4) publications in languages other than English. We included and pooled results for patients with HFrEF or HFpEF, as the magnitude and implications of HF on V̇O₂peak are considered relatively consistent between HF phenotypes [4, 7].

Study quality was assessed using the appraisal tool for cross-sectional studies (AXIS). This validated instrument consists of 20 criteria specifically designed to evaluate the quality of cross-sectional studies, with a maximum score of 20 indicating the highest quality [8]. For the primary outcome, we assessed the presence of study bias using funnel plots by comparing the studies’ mean differences (MD) with their standard errors (SE) and performed Egger’s test to evaluate funnel plot symmetry [9]. The review was not pre-registered, and a study protocol was not prepared.

### Data analysis

The primary outcome was V̇O₂peak (reported as absolute values or indexed to body weight), while the secondary outcomes included the underlying physiologic determinants of V̇O₂peak according to the Fick principle (peak exercise cardiac output, stroke volume, heart rate, and arterio-venous oxygen content difference, a-vO_2_diff) in absolute values, or indexed to body surface area where relevant and reported. Differences in primary and secondary outcomes between HF and CON group (aim 1) and between YO vs. MO and MO vs. OO (aim 2) were quantified using meta-analysis based on random-effects models. Using these models, the weighted average effect size, defined as the weighted mean difference (WMD) with 95% confidence intervals (CI), was calculated for each outcome measure. Each study’s weight was determined by the inverse of its variance, meaning that studies with larger sample sizes and lower variance were given more weight. We conducted subgroup meta-regression analysis to investigate potential differences in relative V̇O₂peak and heart rate (HR) between HF phenotypes and beta blocker use as part of aim 1. Heterogeneity among effect sizes was assessed using *I*^2^ and *τ*^2^ statistics. Forest plots were created to illustrate individual effect sizes, confidence intervals, and the associated *p* value for hypothesis testing at the alpha level of 0.05. All analyses were conducted with the R metacont package (R Core Team 2016, R Foundation for Statistical Computing, Vienna, Austria).

## Results

**Fig. 1:**
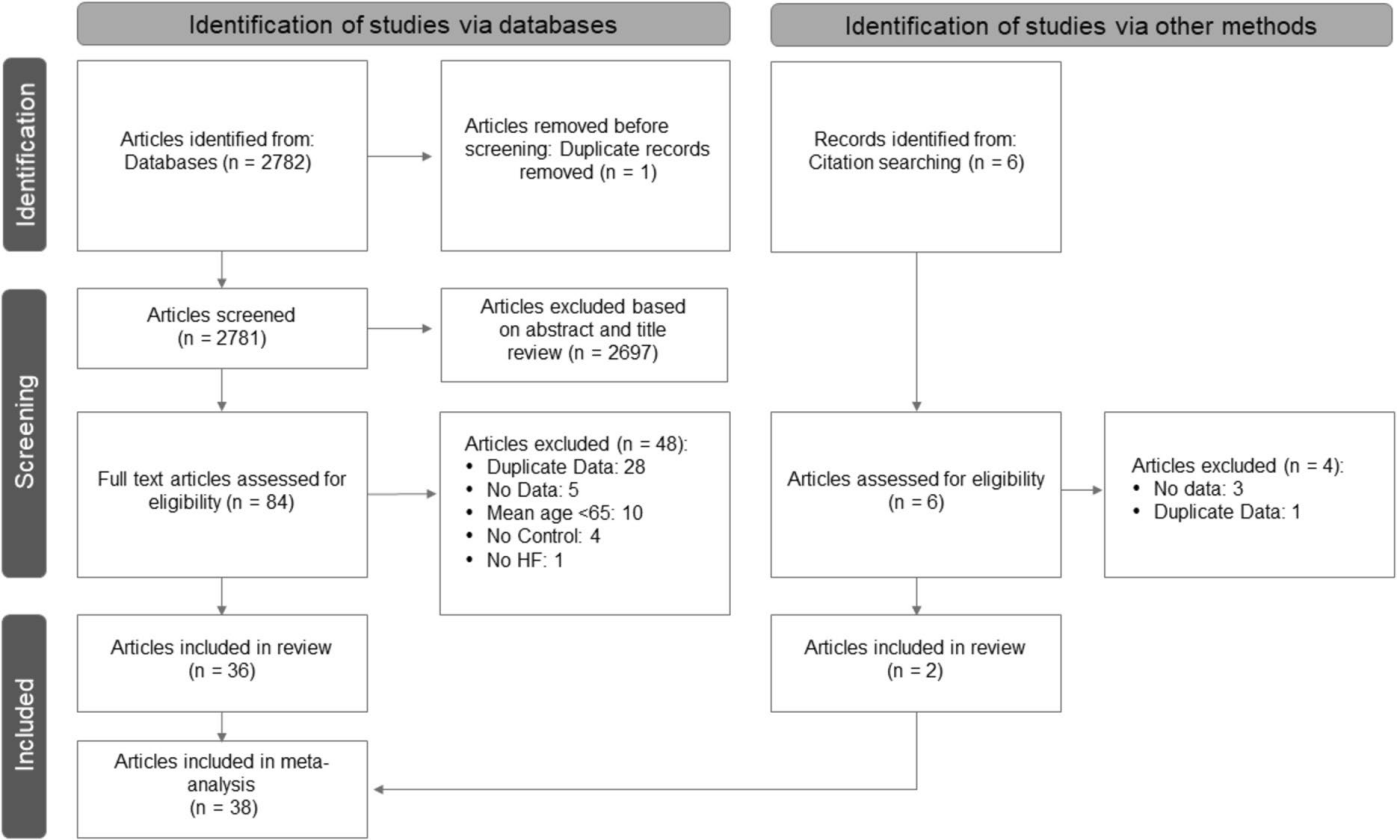
PRISMA diagram for selection of studies included in the meta-analysis

Our initial search yielded 2782 records of which 38 unique studies met the inclusion criteria and were included in this analysis (Fig. 1). Specifically, 30 studies met our aim 1 inclusion criteria (HF,* n* = 1093, mean age 70 years, 46% female; mean body mass 77 kg, 53% HFrEF, vs. 47% HFpEF; CON, *n* = 942, mean age 69 years, 52% female, mean body mass 72 kg (Table 1), while eight studies met aim 2 inclusion criteria comparing 3420 YO (60–69 years) with 2457 MO (70–79 years), and 877 MO with 271 OO (≥ 80 years) individuals with HF (Table 2). Cardiopulmonary exercise testing was performed on a cycle ergometer (CYC) (*n* = 15 studies), treadmill (TM) (*n* = 13 studies) or combination of CYC or TM (*n* = 2 studies) to quantify V̇O₂peak. Finally, in the studies that measured the Fick determinants of V̇O₂peak in aim 1, cardiac output and stroke volume were measured using acetylene rebreathing (*n* = 3) [10–12], impedance cardiography (*n* = 2) [13, 14], or echocardiography (*n* = 1) [15] techniques, while in all studies, a-vO_2_diff was back-calculated from V̇O₂peak and peak cardiac output using the Fick equation (a-VO₂ diff = absolute V̇O₂peak ÷ peak cardiac output) [10, 11, 13, 14].

**Table 1:** Demographic data – Patients with heart failure (HF; mean age ≥ 65 years) vs. CON

Author	Group	No. (% female)	Age (year)	Body weight (kg)	BMI (kg/m^2^)	Comorbidities (%)	Ejection fraction (%)	Medications for HF population (%)
Peeters 1996 [16]	CON HF	11 (64) 10 (70)	80 79	NR NR	NR NR	CON: NR HF: NR	NR 30	CON: NR HF: NR
Toth 1997 [17]	CON HF	52 (4) 14 (0)	69 67	80 83	NR NR	CON: NR HF: NR	NR 23	CON: NR HF: NR
Kitzman 2002 [18]	CON HF	28 (61) 60 (35)	68 70	70 78	24 26	CON: NR HF: HTN (48) DM (27)	54 31	CON: NR HF: BBs (17) ACEi/ARBs (83) CCBs (24) Diu (88)
Nishio 2003 [19]	CON HF	69 (100) 75 (100)	72.0 73.6	53 54	NR NR	CON: NR HF: NR	68 60	CON: NR HF: NR
Witte 2004 [20]	CON HF	58 (48) 173 (30)	70.3 72.5	NR NR	NR NR	CON: NR HF: NR	57 31	CON: BBs (7) ACEi/ARBs (2) Diu (7) HF: BBs (51) ACEi (72)/ARBs (5) Diu (6)
Borlaug 2006 [21]	CON HF	19 (84) 17 (94)	65 65	NR NR	31.3 36.8	CON: NR HF: HTN (100) OB (76)	NR NR	CON: BBs (58) ACEi/ARBs (50) CCBs (22) Diu (33) HF: BBs (65) ACEi/ARBs (76) CCBs (18) Diu (100)
Miller 2009 [22]	CON HF	10 (40) 10 (30)	69.3 72.2	82 92	NR NR	CON: NR HF: HTN (100) DM (30)	NR HFrEF (26) HFpEF (47)	CON: NR HF: NR
Munkvik 2010 [23]	CON HF	13 (0) 11 (0)	67.7 68.4	NR NR	27.7 27.7	CON: NR HF: NR	68 29	CON: BBs (8) ACEi (0)/ARBs (15) HF: BBs (100) ACEi (64)/ARBs (36)
Phan 2010 [24]	CON HF	41 (63) 41 (71)	67 69	NR NR	26 31	CON: NR HF: HTN (68) OB (85) DM (7)	64 64	CON: NR HF: ACEi (44)/ARBs (17) CCBs (34) Diu (22)
Beale 2011 [25]	CON HF	7 (43) 10 (2)	67 75	79 86	NR NR	CON: NR HF: NR	69 36	CON: NR HF: BBs (90) ACEi (40)/ARBs (40) CCBs (20) Diu (80)
Bhella 2011 [10]	CON HF	13 (46) 11 (64)	70.2 73.0	73 89	25.7 33.6	CON: NR HF: HTN (100) DM (55)	NR NR	CON: NR HF: BBs (55) ACEi/ARBs (82) CCBs (45) Diu (91)
Fu 2011 [13]	CON HF	39 (31) 48 (29)	67.2 67.5	62 63	23.9 24.7	CON: NR HF: NR	NR 38	CON: BBs (8) ACEi/ARBs (5) CCBs (8) Diu (0) HF: BBs (92) ACEi/ARBs (96) CCBs (13) Diu (67)
Savage 2011 [26]	CON HF	11 (45) 10 (30)	72.1 73.4	NR NR	NR NR	CON: HTN (45) HF: DM (30)	62 32	CON: ACEi/ARBs (18) Diu (27) HF: BBs (90) ACEi/ARBs (100) Diu (50)
Sandri 2012 (a) [27]	CON HF	15 (20) 15 (20)	72 72	NR NR	28 28	CON: HTN (87) DM (47) HF: HTN (93) DM (60)	62 29	CON: BBs (80) ACEi (53)/ARBs (20) Diu (13) HF: BBs (100) ACEi (93)/ARBs (7) Diu (80)
Sandri 2012 (b) [27]	CON HF	15 (20) 15 (20)	72 72	NR NR	28 28	CON: HTN (80) DM (27) HF: HTN (93) DM (60)	62 28	CON: BBs (73) ACEi (60)/ARBs (20) Diu (20) HF: BBs (100) ACEi (87)/ARBs (13) Diu (93)
Rullman 2013 [28]	CON HF	7 (71) 8 (20)	71 65	NR NR	NR NR	CON: HTN (29) DM (0) HF: HTN (25) DM (38)	61 24	CON: BBs (14) ACEi/ARBs (29) Diu (0) HF: BBs (88) ACEi/ARBs (100) Diu (100)
Tan 2013 [29]	CON HF	38 (76) 67 (67)	71 73	NR NR	24 30	CON: DM (24) HF: DM (24)	63 61	CON: NR HF: BBs (37) ACEi (38)/ARBs (27) CCBs (27) Diu (49)
Zavin 2013 [30]	CON HF	39 31	65.3 67.4	87 86	30.2 28.9	CON: HTN (52) DM (35) HF: HTN (61) DM (48)	NR 29	CON: BBs (5) ACEi (15)/ARBs (5) Diu (13) HF: BBs (81) CEi (84)/ARBs (6) Diu (65)
Kitzman 2014 [31]	CON HF	43 (51) 22 (82)	69.2 69.8	78 80	26.7 29.7	CON: NR HF: HTN (73) DM (14)	65 64	CON: NR HF: BBs (36) ACEi/ARBs (0) CCBs (23) Diu (55)
Olson 2016 [15]	CON HF	26 (69) 20 (75)	65 67	NR NR	29.1 34.5	CON: HTN (62) HF: HTN (85)	58 60	CON: Diu (0) HF: Diu (60)
Tarperi 2016 [32]	CON HF	11 (0) 9 (0)	67 66	80 75	27.6 25.8	CON: NR HF: NR	NR 32	CON: NR HF: BBs (100) ACEi/ARBs (100)
Thompson 2017 [33]	CON HF	16 (38) 23 (22)	67 66	79 89	26.9 29.7	CON: NR HF: NR	63 38	CON: NR HF: NR
Kondo 2018 [34]	CON HF**	23 (30) 65 (17)	66 68	NR NR	23.4 23.6	CON: HTN (52) DM (17) HF: HTN (66) DM (57)	64 42	CON: BBs (65) ACEi/ARBs (74) Diu (13) HF: BBs (89) ACEi/ARBs (83) Diu (66)
Mahmod 2018 [35]	CON HF	14 (57) 27 (67)	69 72	NR NR	26 29	CON: HTN (0) HF: HTN (44)	NR NR	CON: NR HF: BBs (11) ACEi/ARBs (52) Diu (44)
Mordi 2018 [36]	CON HF	28 (50) 62 (68)	67.7 70.8	NR NR	25.6 29.2	CON: HTN (0) DM (0) HF: HTN (76) DM (10)	64 65	CON: BBs (0) ACEi/ARBs (0) CCBs (0) Diu (0) HF: BBs (35) ACEi/ARBs (50) CCBs (34) Diu (48)
Hearon 2019 [11]	CON HF	18 (56) 18 (56)	70 68	75 99	26 35	CON: NR HF: NR	NR NR	CON: NR HF: BBs (61) ACEi/ARBs (61) Diu (89)
Parovic 2019 [14]	CON HF	20 (35) 17 (24)	65 67	76 84	27.7 28.2	CON: NR HF: NR	NR 32	CON: NR HF: BBs (100) ACEi (88)/ARBs (12) Diu (76)
Rullman 2020 [37]	CON HF	28 (71) 66 (20)	69.2 69.0	NR NR	26.1 27.6	CON: HTN (64) DM (11) HF: HTN (58) DM (44)	58 26	CON: BBs (29) ACEi (18)/ARBs (25) Diu (29) HF: BBs (95) ACEi (65)/ARBs (33) Diu (94)
Sarma 2021 [12]	CON HF	14 (50) 20 (60)	72 69	NR NR	27 35.1	CON: HTN (0) DM (0) HF: HTN (95) DM (65)	NR NR	CON: BBs (0) ACEi/ARBs (0) CCs (0) Diu (0) HF: BBs (90) ACEi/ARBs (65) CCs (30) Diu (95)
Saito 2023 [38]	CON HF	179 (57) 83 (55)	66 74	NR NR	24 23.9	CON: HTN (69) DM (18) HF: HTN (79) DM (22)	63 63	CON: BBs (15) ACEi/ARBs (32) Diu (9) HF: BBs (29) ACEi/ARBs (49) Diu (28)
Scandalis 2023 [39]	CON HF	45 (80) 27 (85)	70.2 68.4	74 105	26.8 38.9	CON: NR HF: HTN (100) DIA (37)	59 61	CON: NR HF: BBs (44) ACEi (37)/ARBs (41) CCBs (22) Diu (93)

Data are presented as mean unless otherwise specified. Abbreviations: *ACEi*, angiotensin-converting enzyme inhibitors; *ARBs*, angiotensin II receptor blockers; *BBs*, beta blockers; *CCBs*, calcium channel blockers; *CON*, control group; *DM*, diabetes mellitus; *Diu*, diuretics; *HF*, heart failure; *HTN*, hypertension; *OB*, obesity

**Table 2:** HF older age subgroups: YO, MO, and OO

Author	Group	Number (% female)	Age (year)	Body weight (kg)	BMI (kg/m^2^)	Comorbidities (%)	Ejection fraction (%)	Medications (%)
Scardovi 2007 [40]	MO OO	297 (NR) 98 (NR)	NR 83	NR NR	NR NR	*Total cohort* DM (11)	NR NR	*Total cohort* BBs (39) ACEi/ARBs (57)
Williams 2007 [41]	YO MO	7 (NR) 6 (NR)	67 74	88 80	30 27	YO: NR MO: NR	26 27	YO: BBs (71) ACEi (86)/ARBs (14) CCBs (14) Diu (86) MO: BBs (33) ACEi (67)/ARBs (33) CCBs (0) Diu (100)
Forman 2009 [42]	YO MO	640 (26) 477 (21)	64.6 75.5	89 82	29 27	YO: DM (38) MO: DM (31)	25 26	YO: BBs (94) ACEi/ARBs (94) MO: BBs (91) ACEi/ARBs (92)
Ciolac 2013 [43]	YO MO	75 (20) 18 (33)	60–69 70–79	NR NR	25 27	YO: NR MO: NR	30 25	YO: BBs (100) ACEi/ARBs (79) Diu (72) MO: BBs (100) ACEi/ARBs (88) Diu (77)
Carubelli 2015 [44]	YO MO	1080 (16) 990 (18)	65 74.3	75 74	26 26	YO: NR MO: NR	30 34	YO: BBs (81) ACEi (76)/ARBs (17) Diu (80) MO: BBs (76) ACEi (70)/ARBs (20) Diu (85)
Nanayakkara 2017 [45]	YO MO	21 (57) 19 (42)	62 75	90 83	32 30	YO: NR MO: NR	NR NR	YO: NR MO: NR
Kato 2018 (a) [46]	YO MO	146 (NR) 56 (NR)	62 76	NR NR	24 22	YO: HTN (53) DM (49) MO: HTN (66) DM (38)	27 28	YO: BBs (67) MO: BBs (70)
Kato 2018 (b) [46]	YO MO	61 (NR) 26 (NR)	63 76	NR NR	24 22	YO: HTN (66) DM (36) MO: HTN (65) DM (23)	45 45	YO: BBs (49) MO: BBs (54)
Kato 2018 (c) [46]	YO MO	494 (NR) 285 (NR)	63 76	NR NR	23 22	YO: HTN (54) DM (28) MO: HTN (48) DM (32)	66 68	YO: BBs (34) MO: BBs (35)
Peterman 2021 (a) [47]	YO MO OO	477 (NR) 290 (NR) 85 (NR)	60–69 70–79 ≥ 80	NR NR NR	NR NR NR	YO: NR MO: NR OO: NR	NR NR NR	YO: NR MO: NR OO: NR
Peterman 2021 (b) [47]	YO MO OO	138 (NR) 57 (NR) 11 (NR)	60–69 70–79 ≥ 80	NR NR NR	NR NR NR	YO: NR MO: NR OO: NR	NR NR NR	YO: NR MO: NR OO: NR
Peterman 2021 (c) [47]	YO MO OO	219 (NR) 176 (NR) 56 (NR)	60–69 70–79 ≥ 80	NR NR NR	NR NR NR	YO: NR MO: NR OO: NR	NR NR NR	YO: NR MO: NR OO: NR
Peterman 2021 (d) [47]	YO MO OO	62 (NR) 57 (NR) 21 (NR)	60–69 70–79 ≥ 80	NR NR NR	NR NR NR	YO: NR MO: NR OO: NR	NR NR NR	YO: NR MO: NR OO: NR

Data are presented as mean unless otherwise specified. Abbreviations: *ACEi*, angiotensin-converting enzyme inhibitors; *ARBs*, angiotensin II receptor blockers; *BBs*, beta blockers; *CCBs*, calcium channel blockers; *CON*, control group; *DM*, diabetes mellitus; *Diu*, diuretics; *HF*, heart failure; *HTN*, hypertension; *OB*, obesity

**Fig. 2:**
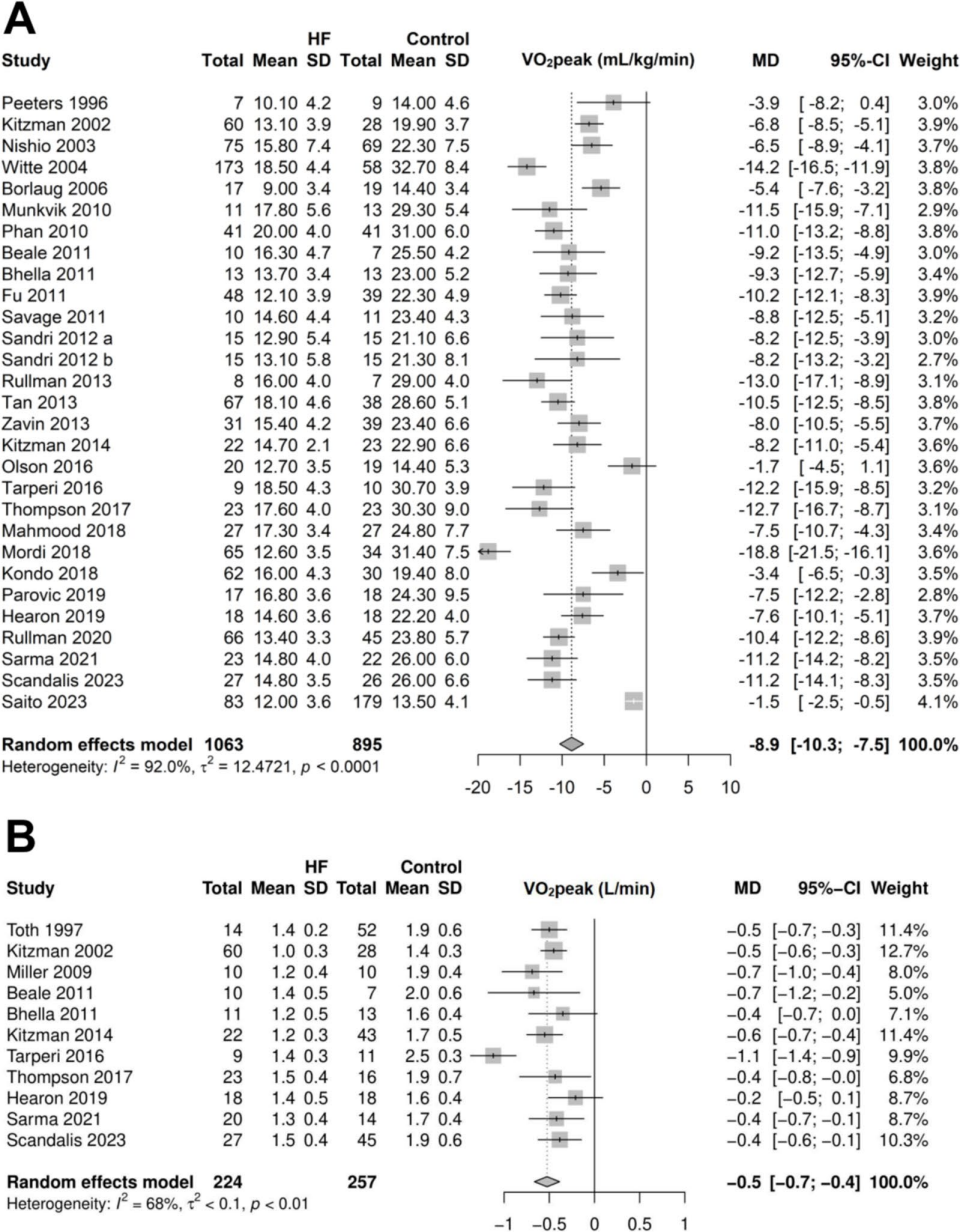
Peak oxygen uptake in older patients with heart failure (HF) versus healthy controls. Forest plots showing **A** peak oxygen uptake (V̇O₂peak) indexed to body weight (mL/kg/min) and **B** as absolute values in older patients with HF versus non-HF controls derived from a random-effects meta-analysis. CI, confidence interval; MD, mean difference; SD, standard deviation

V̇O₂peak was significantly lower in HF compared to CON (Fig. 2) when measured in absolute values (WMD − 0.5 L/min, [95% CI] − 0.7 to − 0.4 L/min, *I*^2^ = 68%, *n* = 481) or when indexed to body mass (WMD − 8.8 mL/kg/min, [95% CI] − 10.3 to − 7.3 mL/kg/min, *I*^2^ = 92%, *n* = 1944). Meta-regression analyses revealed no significant differences in V̇O₂peak WMD (*β* = 0.40 mL/kg/min, *p* = 0.774) when comparing HFrEF and HFpEF patients to their respective controls. A further meta-regression on beta-blocker use also showed no significant effect on V̇O₂peak (*β* = − 2.64 mL/kg/min, *p* = 0.177).

In the studies that measured the Fick determinants of V̇O₂peak, peak exercise cardiac output (WMD − 2.6 L/min, [95% CI] − 4.4 to − 0.9 L/min, *I*^2^ = 79%, *n* = 264), cardiac index (WMD − 1.4 L/min/m^2^, [95% CI] − 2.2 to − 0.6 L/min/m^2^, *I*^2^ = 50%, *n* = 131), and HR were significantly lower in HF compared to CON (WMD − 22.7 bpm, [95% CI] − 28.9 to − 16.5 bpm, *I*^2^ = 84%, *n* = 1172). However, no significant difference in HR was observed between HFpEF and HFrEF compared to their respective controls (*β* = 2.18 bpm, *p* = 0.706), nor with beta-blocker use (*β* = − 14.29 bpm, *p* = 0.146). No significant difference in peak exercise stroke volume (WMD 2.5 mL, [95% CI] − 11.9 to 16.9 mL, *I*^2^ = 92%, *n* = 227), peak exercise systolic blood pressure (WMD − 10.3 mmHg, [95% CI] − 22.0 to − 1.4 mmHg, *I*^2^ = 91%, *n* = 959), peak exercise diastolic blood pressure (WMD − 3.1 mmHg, [95% CI] − 8.8 to 2.6 mmHg, *I*^2^ = 88%, *n* = 593), or a-vO_2_ diff (WMD − 2.8 mL/dL, [95% CI] − 5.9 to 0.2 mL/dL, *I*^2^ = 96%, *n* = 218, Supplementary material, Fig. 1) was found between groups. Notably, the WMDs for V̇O₂peak in HF vs CON in the sub-group of studies that measured Fick determinants (WMD’s − 9.2 to − 7.9 mL/kg/min) were similar to that measured in the analysis for aim 1 (− 8.8 mL/kg/min).

**Fig. 3:**
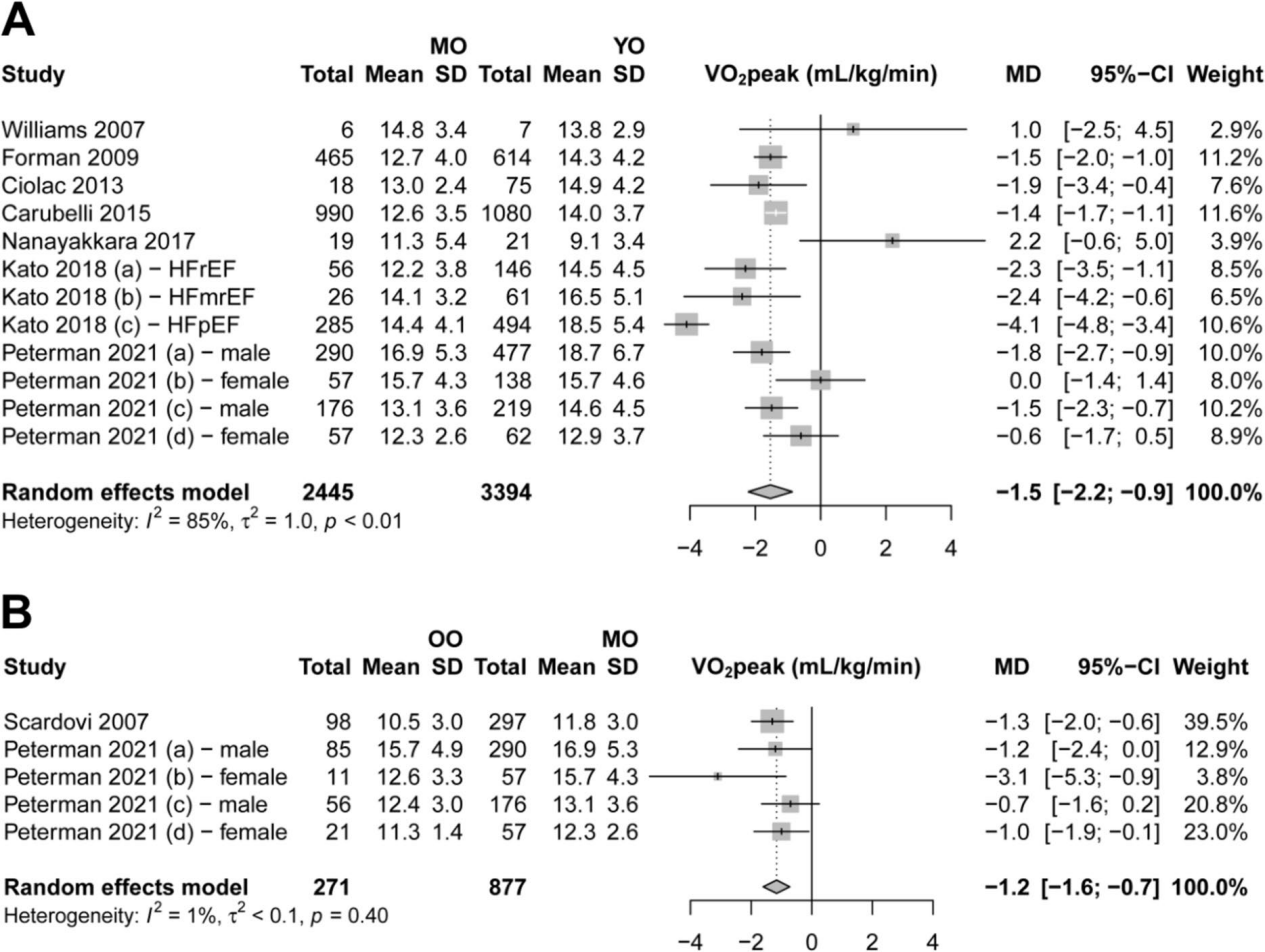
Peak oxygen uptake in patients with heart failure (HF) across the older age continuum. Forest plots showing peak oxygen uptake (V̇O₂peak) indexed to body weight (mL/kg/min) in **A** young-old (YO, 60–69 years) versus middle-old (MO, 70–79 years) patients with HF and **B** MO versus old-old (OO, ≥ 80 years) patients with HF. Results are derived from a random effects meta-analysis. Abbreviations: CI, confidence interval; MD, mean difference; SD, standard deviation

**Fig. 4:**
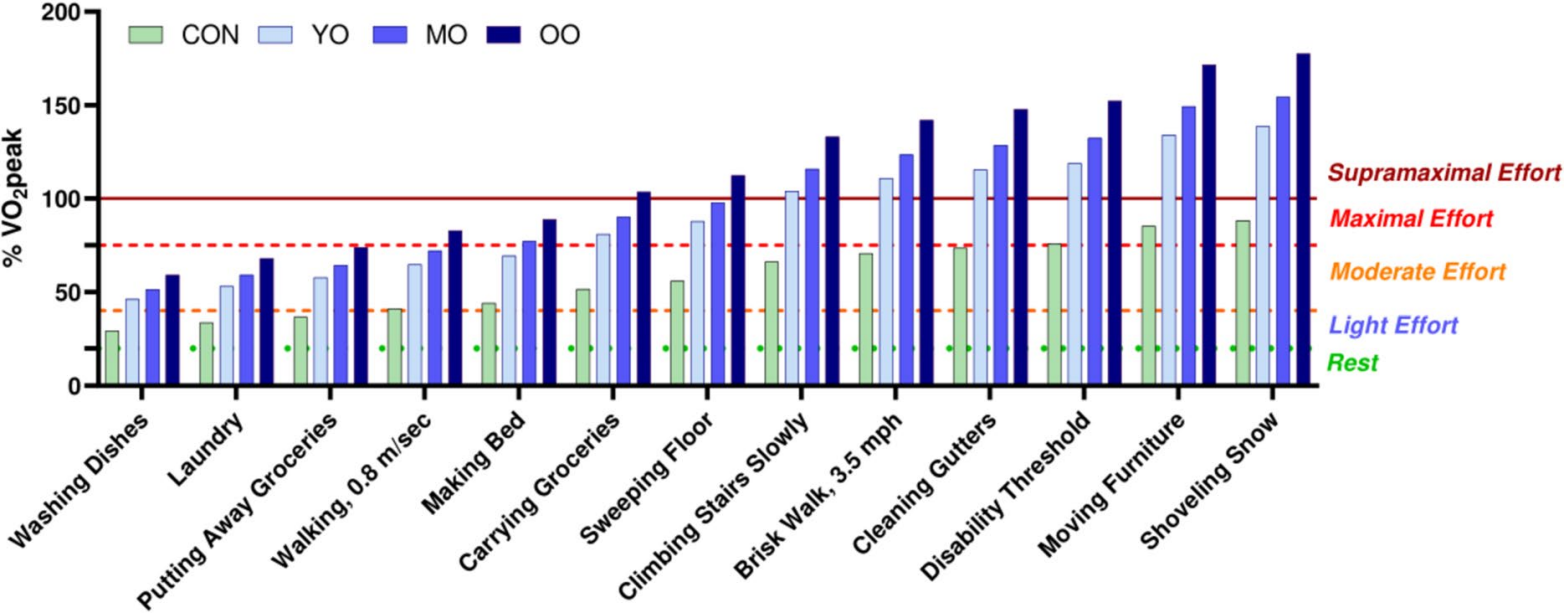
Peak oxygen uptake, activities of daily living and functional independence with heart failure (HF) and aging. Weighted mean V̇O₂peak values for each study group are expressed as a relative percentage of the metabolic requirements for activities of daily living (from the compendium of physical activities [48]) and the threshold for functional independence (V̇O₂peak of 18.0 mL/kg/min [49]). Represented study groups are older controls (CON, mean age 69 years; aim 1) and young-old (YO, 60–69 years), middle-old (MO, 70–79 years), and old-old (OO, ≥ 80 years) patients with HF from aim 2

Regarding aim 2, MO HF patients had a significantly lower V̇O₂peak indexed to body weight (WMD − 1.5 mL/kg/min, 95% CI − 2.2 to − 0.9 mL/kg/min, *I*^2^ = 85%, *n* = 5839; Fig. 3) and a lower peak HR (WMD − 8.0 bpm, 95% CI − 10.8 to − 5.2 bpm, *I*^2^ = 72%, *n* = 5839) compared to YO HF patients. A similar trend was seen for V̇O₂peak in OO HF patients compared to MO HF patients (WMD − 1.2 mL/kg/min, 95% CI − 1.6 to − 0.7 mL/kg/min, *I*^2^ = 1%, *n* = 1148), leading to greater limitations in performing daily activities with increasing age (Fig. 4).

The included studies were evaluated for risk of bias using the AXIS tool, resulting in an average score of 14.3 out of 20 points. Key limitations included a lack of sufficient justification for the sample size, inadequate details on the handling of non-respondents (non-response refers to individuals in the study who either do not participate or fail to provide the requested data), and limited transparency regarding funding sources or potential conflicts of interest. Full AXIS scores for each study are available in Supplementary material, Table 1. Funnel plot analysis for the primary outcome comparing HF vs. CON revealed asymmetry, with Egger’s test confirming potential publication bias through a significant intercept of − 4.35 (*p* = 0.001). No significant asymmetry was found when comparing YO vs. MO and MO vs. OO (Supplementary material, Fig. 3A–C).

## Discussion

To our knowledge, this is the first systematic review and meta-analysis to assess V̇O₂peak in older individuals with HF. Our analysis yielded three major novel key findings. First, older HF patients have a significantly lower in V̇O₂peak (~ 8.8 mL/kg/min) compared to CON. Second, in the studies that measured the Fick determinants, the reduction in V̇O₂peak was primarily the result of a lower peak exercise HR (23 bpm), cardiac output, and cardiac index (− 2.6 L/min and − 1.4 L/min/m^2^, respectively). Lastly, across the older age HF continuum, V̇O₂peak was − 1.5 mL/kg/min lower from the YO to MO groups, and − 1.2 mL/kg/min lower from the MO to OO groups.

The results of our analysis emphasize the significant reduction in V̇O₂peak among older patients with HF and highlight the accelerated physiologic aging associated with HF. Indeed, the mean values for the HF cohort (13.5 mL/kg/min) at 69 years of age are equivalent to what would be expected in healthy older adults in their eighth decade of life [49]. Secondly, the weighted mean values observed in the older HF cohorts are 14% lower for YO, 26% lower for MO, and 30% lower for OO than the metabolic requirements for full and independent living (V̇O₂peak of 18.0 mL/kg/min [49]). This is a result of the metabolic requirements for many activities of daily living requiring maximal [48], if not supramaximal, effort as reduced V̇O₂peak is a result of both HF and aging. In contrast, the mean V̇O₂peak measured in CON is above the threshold for independent living, and as such, is sufficient to complete most daily tasks. As such, this severely restricts the ability to perform everyday tasks such as cleaning, shopping, and climbing stairs, and explains the pronounced disability that may be experienced by older individuals with HF. Notably, the extremely low V̇O₂peak in the OO group indicates that walking at 0.8 m/s (a value predictive of markedly increased risk of frailty and mortality) requires almost maximal effort, exemplifying the pathway through which HF and older age intersect to accelerate frailty and disability [50, 51]. Indeed, the loss of mobility and independence associated with reduced V̇O₂peak increases the need for assistance and care among older patients with HF, placing a substantial burden not only on the patients themselves and their family members, but also on the healthcare system [52]. The progressive, age-related reduction within the HF groups is consistent with expected age-related reductions in V̇O₂peak among individuals without HF [53, 54], but when combined with the overall decrement in V̇O₂peak associated with HF itself resulted in extremely low V̇O₂peak values in the OO, significantly increasing their risk of disability, frailty, and adverse clinical outcomes [55]. These findings highlight the critical importance of understanding the mechanisms driving reduced exercise capacity in advanced age and HF, and the importance of screening for frailty in older HF populations.

The mechanisms underlying the lower V̇O₂peak in older patients with HF have not been well studied, however are likely multifactorial. One of the primary factors is cardiovascular aging, which results in a progressive change in cardiac structure and function, characterized by impaired LV diastolic function (increased chamber stiffness) and left atrial dilation, despite the preservation of left ventricular ejection fraction [56]. In our aim 1 sub-analysis of studies that measured the Fick determinants of V̇O₂peak, older HF patients (≥ 65 years) exhibited a significantly reduced peak cardiac output compared to controls, which is consistent with prior reports identifying the decrease in peak cardiac output as a key driver of age-related declines in V̇O₂peak among healthy volunteers [57]. In turn, the HF-mediated reduction in peak exercise cardiac output was primarily due to a lower peak HR (23 bpm) compared to controls. The mechanism(s) underpinning the reduced HR in older patients with HF are not fully understood, however may be related to age-related downregulation of β-adrenergic receptors, reduced β-adrenergic sensitivity, and a decrease in intrinsic HR, thereby limiting the heart’s ability to exceed its intrinsic rate [58, 59]. This may be further exacerbated in some individuals with HF, due to additional β-adrenergic receptor downregulation and/or desensitization secondary to chronic elevations in sympathetic activity [60]. Indeed, previous studies have confirmed that insufficient cardioacceleration (i.e., chronotropic reluctance and/or incompetence) is a major limiting factor in the exercise capacity of HF patients [21, 61]. The lower maximal HR with age and/or HF may also be related to coronary atherosclerosis and ventricular stiffening, where coronary flow and diastolic filling can be compromised at higher heart rates [62, 63]. Moreover, pulmonary or skeletal muscle dysfunction may also contribute to the lower maximal HR in some individuals, by resulting in premature exercise termination due to fatigue or breathlessness before true maximal HR can be achieved [60]. It is also possible that the use of heart rate–lowering medications (such as beta-blockers) contributes to the reduced peak HR, and potentially to the lower V̇O₂peak, observed in older patients with HF. In our meta-regression analyses, beta-blocker use was associated with lower V̇O₂peak and peak HR, although these associations did not reach statistical significance.

Our analysis found no significant difference in peak stroke volume between older patients with HF and controls, with mixed findings across studies as three reported higher stroke volume in HF patients [10–12] while two reported lower values [13, 15]. These findings may be due to the different techniques used to measure cardiac output, and a focus predominantly on patients with HFpEF. Nevertheless, the variability in stroke volume during peak exercise may reflect a dynamic adaptation, where the lower heart rate in HF patients allowed for increased ventricular filling time, thus sustaining stroke volume and cardiac output [64]. Furthermore, the comparable stroke volume between older patients with HF and CON may be a result of higher use of angiotensin-converting enzyme inhibitors or angiotensin receptor blockers among the HF group, as these medications have been associated with enhanced stroke volume response during exercise [65, 66]. However, as for beta-blocker use, we are unable to determine the impact of medication use on the evaluated outcomes due to insufficient reporting of prescribed medications.

We also found that peak exercise a-vO_2_ diff was lower in older HF patients compared to controls, but this difference did not reach statistical significance. Notably, only five studies measured peak a-vO_2_ diff, and Parovic et al. observed slightly higher oxygen extraction in HF patients compared to controls [14]. Importantly, a-vO_2_ diff is influenced by cardiac output, where decreased output allows more time for O_2_ off-loading and diffusion from red blood cells to skeletal muscle mitochondria, maintaining a-vO_2_ diff despite impairments in microvascular and skeletal muscle oxidative capacity [67, 68]. Therefore, the similar a-vO_2_ diff in the HF group and controls (despite lower cardiac output) suggests impaired O_2_ extraction and diffusion from capillaries to skeletal muscle mitochondria in older HF patients. However, muscle and capillary blood flow heterogeneity, along with a reduced number of oxidative muscle fibers and/or impaired mitochondrial function, may also lead to lower O_2_ extraction during exercise [69].

Decrements in V̇O₂peak are closely linked to worsening clinical outcomes, particularly in oldest-old HF patients. Indeed, a retrospective cohort study by Garred et al. found that, while mortality decreased from 66 to 43% across all age groups, the smallest reduction was observed in those aged ≥ 80 years, who had the highest initial risk [70]. Despite advances in curative treatments extending lifespan, older HF patients often face prolonged disability [71]. Consequently, frailty plays a key role in morbidity and mortality of older HF patients, accelerating disease progression and worsening outcomes. Kitzman et al. found in their REHAB-HF study that among 349 older HF-patients (mean age 73 years), 97% were either frail or pre-frail, and that a 12-week, multi-domain rehabilitation intervention (strength, balance, mobility, and endurance) led to a significant reduction in frailty and improvements in both physical performance and quality of life [72]. Thus, an important goal for older HF patients is to prevent a decline in V̇O₂peak, muscle strength, and physical function in order to be able to function independently in one’s environment. Indeed, even a 1.0 mL/kg/min increase in V̇O₂peak can significantly enhance daily function, reduce hospitalizations, and lower mortality [73].

While our analysis provides valuable insights, several limitations need to be considered. The studies included varied widely in sample sizes, sex distribution, and HF severity and used different cardiopulmonary exercise testing modes, complicating comparisons across findings. Additionally, older, frail patients with severe HF may be underrepresented, as they are less likely to participate in cardiopulmonary exercise testing or studies due to physical limitations. It is possible that HF patients in the oldest age group may have been diagnosed at a later age, resulting in a survivor effect. This phenomenon may overrepresent individuals with better overall health and higher V̇O₂ peak, potentially underestimating the actual V̇O₂peak values associated with aging. Moreover, many studies did not control for key factors such as physical activity, diet, comorbidities, or medication use, which may confound the results and limit our ability to isolate the specific impact of HF on V̇O₂peak. Although we considered beta-blocker use in our analysis, interpretation is limited by reliance on study-level data rather than individual-level comparisons between treated and untreated patients. A further limitation is the presence of asymmetry in the funnel plot for the HF vs. CON comparison, indicating potential publication bias, which could affect the reliability of the pooled estimate. Finally, although only six studies measured peak exercise cardiac output and five reported a-vO_2_ diff, the sample size for the former (*n* = 264) and the latter (*n* = 228) is the largest reported for older HF patients.

In conclusion, our finding of a significant and marked reduction in V̇O₂peak observed in older patients with HF underscores a profound impairment in physiologic reserve, directly linked to their decreased cardiac function−with significant implications for their risk of disability and frailty. This impairment becomes more pronounced with advancing age, with V̇O₂peak progressively diminishing across the age spectrum from YO to OO HF patients. This novel finding highlights the urgent need for effective interventions, such as exercise and/or functional training, to enhance physiologic reserve and exercise capacity across all the older HF age continuum. Future studies should take a comprehensive approach, examining both cardiac and peripheral factors while also exploring the potential impact of comorbidities, medications, and physical activity levels. Such strategies may prove to benefit functional independence and quality of life in those living with HF.

## Supplementary Information

Below is the link to the electronic supplementary material.

Supplementary file1 (PDF 2.19 MB)

## Data Availability

Not applicable.
