# Supplementary material for: Peak oxygen uptake in older adults with heart failure: a systematic review and meta-analysis
Source: GeroScience. 2025 Jul 28;48(3):4827–45. doi: 10.1007/s11357-025-01795-3 (PMC13356004; doi:10.1007/s11357-025-01795-3)
Supplement: Supplementary file 1 — Supplementary file1 (PDF 2.19 MB) [file 11357_2025_1795_MOESM1_ESM.pdf]

Supplemental Materials

Title: Peak Oxygen Uptake in Older Adults with Heart Failure: A Systematic Review and Meta-Analysis

Veronika Schmid[1,2], Sarah Paterson[2], Christopher Weinkauf[2], Jing Wang[3], Corey R. Tomczak[4], David Niederseer[5,6,7], Jan Vontobel[5], Daniel E. Forman[8,9], Martin Halle[1], Michael D. Nelson[10], Stephen J. Foulkes[2,11*], Mark J. Haykowsky[2*]

[*], Contributed equally as senior authors

[1] Department of Preventive Sports Medicine and Sports Cardiology, School of Medicine and Health, TUM University Hospital, Technical University of Munich (TUM), Munich, Bavaria, Germany.

[2] Integrated Cardiovascular Exercise Physiology and Rehabilitation Lab, Faculty of Nursing, College of Health Science, University of Alberta, Edmonton, Alberta, Canada.

[3] Division of Public Health, School of Medicine, University of Utah, Salt Lake City, Utah, USA.

[4] College of Kinesiology, University of Saskatchewan, Saskatoon, Saskatchewan, Canada.

[5] Hochgebirgsklinik Davos, Medicine Campus Davos, Davos, Switzerland.

[6] Christine Kuehne Center for Allergy Research and Education (CK-CARE), Medicine Campus Davos, Davos, Switzerland.

[7] Department of Cardiology, Center of Translational and Experimental Cardiology (CTEC), University Heart Center Zurich, University Hospital Zurich, University of Zurich, Zurich, Switzerland.

[8] Geriatric Research, Education, and Clinical Center (GRECC), VA Pittsburgh Healthcare System, Pittsburgh, Pennsylvania, USA.

[9] Department of Medicine, Divisions of Geriatrics and Cardiology, University of Pittsburgh, Pittsburgh, Pennsylvania, USA.

[10] Department of Kinesiology, University at Texas Arlington, Arlington Texas, USA.

[11] Heart, Exercise and Research Trials Lab, St Vincent's Institute of Medical Research, Fitzroy, Victoria, Australia.

Table of Contents

Supplementary Figure 1. Cardiac Output (L/min; Panel A), Cardiac Index (L/min/m2; Panel B), Stroke Volume (mL; Panel C), Heart Rate (b/min; Panel D), C(a-v)O2 Diff (mL/dL; Panel E) in heart failure (HF) and controls (CON).

Supplementary Figure 2. Systolic Blood pressure (SBP) (mmHg; Panel A) and Diastolic Blood pressure (DBP) (mmHg; Panel B) in HF and CON.

Supplementary Figure 3. Funnel plots and Egger´s Test for VO2peak (mL/min/kg) in HF vs. CON (Panel A); Young-Old (YO) vs. Middle-Old (MO) HF (Panel B); MO vs. Oldest Old (OO) HF (Panel C).

Supplementary Table 1. Results of study quality assessment using the AXIS Tool.

Supplement Figure 1. Cardiac Output (L/min; Panel A), Cardiac Index (L/min/m^2; Panel B), Stroke Volume (mL; Panel C), Heart Rate (b/min; Panel D), C(a-v)O_2 Diff (mL/dL; Panel E) in heart failure (HF) and controls (CON).

A

Study	HF Total	HF Mean	HF SD	Control Total	Control Mean	Control SD	MD	95%-CI	Weight
Bhella 2011	11	15.10	4.3	13	16.40	4.1	-1.3	[-4.7; 2.1]	12.5%
Fu 2011	48	7.60	2.1	39	12.10	1.9	-4.5	[-5.3; -3.7]	22.8%
Olson 2016	20	9.20	2.5	26	13.20	3.7	-4.0	[-5.8; -2.2]	19.1%
Hearon 2019	18	13.90	3.5	13	13.40	4.0	0.5	[-2.2; 3.2]	15.1%
Parovic 2019	17	13.50	4.0	18	18.20	6.3	-4.7	[-8.2; -1.2]	12.1%
Sarma 2021	20	11.20	2.8	14	12.40	2.9	-1.2	[-3.2; 0.8]	18.4%
**Random effects model**	**134**			**123**			**-2.7**	**[-4.4; -1.0]**	**100.0%**

Heterogeneity: I^2 = 76.6%, τ^2 = 3.1624, p = 0.0007

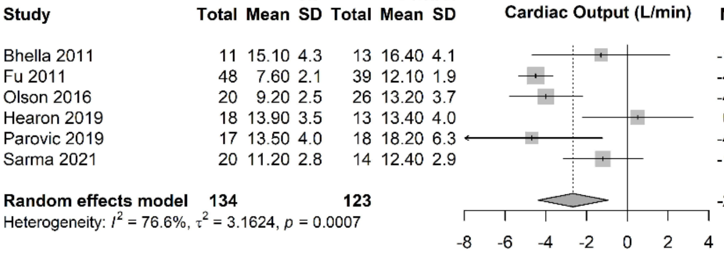

B

Study	HF Total	HF Mean	HF SD	Control Total	Control Mean	Control SD	MD	95%-CI	Weight
Bhella 2011	11	7.60	1.8	13	8.80	1.6	-1.2	[-2.6; 0.2]	20.4%
Hearon 2019	18	6.60	1.5	18	7.20	1.8	-0.6	[-1.7; 0.5]	26.5%
Parovic 2019	17	6.90	1.7	20	9.80	2.9	-2.9	[-4.4; -1.4]	18.2%
Sarma 2021	20	5.20	1.0	14	6.60	1.2	-1.4	[-2.2; -0.6]	35.0%
**Random effects model**	**66**			**65**			**-1.4**	**[-2.2; -0.6]**	**100.0%**

Heterogeneity: I^2 = 49.8%, τ^2 = 0.3217, p = 0.1130

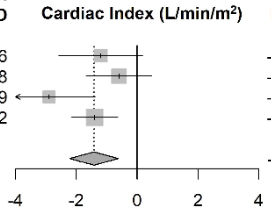

C

Study	HF Total	HF Mean	HF SD	Control Total	Control Mean	Control SD	MD	95%-CI	Weight
Bhella 2011	11	113.0	18.0	13	102.0	24.0	11.0	[−5.8; 27.8]	18.1%
Fu 2011	48	58.0	21.8	39	72.0	12.5	−14.0	[−21.3; −6.7]	22.6%
Olson 2016	20	80.0	25.1	26	91.6	22.1	−11.6	[−25.5; 2.3]	19.6%
Hearon 2019	18	104.0	15.0	18	85.0	25.0	19.0	[5.5; 32.5]	19.9%
Sarma 2021	20	92.0	18.0	14	81.0	21.0	11.0	[−2.5; 24.5]	19.8%
**Random effects model**	**117**			**110**			**2.5**	**[−11.9; 16.9]**	**100.0%**

Heterogeneity: I^2 = 85%, τ^2 = 226.2, p < 0.01

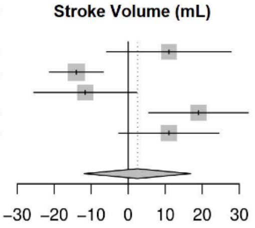

D

Study	HF Total	HF Mean	HF SD	Control Total	Control Mean	Control SD	MD	95%-CI	Weight
Peeters 1996	7	111.0	21.0	9	128.0	24.0	−17.0	[−39.1; 5.1]	3.8%
Kitzman 2002	60	127.0	25.6	28	143.0	23.8	−16.0	[−26.9; −5.1]	6.0%
Borlaug 2006	17	87.0	20.0	19	115.0	22.0	−28.0	[−41.7; −14.3]	5.5%
Phan 2010	41	139.0	22.0	41	171.0	18.0	−32.0	[−40.7; −23.3]	6.5%
Bhella 2011	11	133.0	27.0	13	157.0	19.0	−24.0	[−43.0; −5.0]	4.4%
Fu 2011	48	115.0	14.6	39	153.0	12.5	−38.0	[−43.7; −32.3]	7.0%
Zavin 2013	31	105.7	20.4	39	133.9	19.0	−28.2	[−37.5; −18.9]	6.4%
Kitzman 2014	22	134.0	16.0	43	148.0	16.0	−14.0	[−22.2; −5.8]	6.6%
Olson 2016	20	119.0	22.0	26	144.0	20.0	−25.0	[−37.3; −12.7]	5.8%
Thompson 2017	23	124.0	30.0	16	146.0	24.0	−22.0	[−39.0; −5.0]	4.8%
Kondo 2018	65	124.0	25.0	23	132.0	19.0	−8.0	[−17.9; 1.9]	6.3%
Mordi 2018	62	125.9	25.0	28	149.6	12.6	−23.7	[−31.5; −15.9]	6.7%
Hearon 2019	18	132.0	23.0	18	158.0	13.0	−26.0	[−38.2; −13.8]	5.8%
Parovic 2019	17	111.0	20.9	20	148.0	23.7	−37.0	[−51.4; −22.6]	5.3%
Sarma 2021	20	122.0	20.0	14	155.0	14.0	−33.0	[−44.4; −21.6]	5.9%
Saito 2023	83	113.0	25.0	179	115.0	20.0	−2.0	[−8.1; 4.1]	6.9%
Scandalis 2023	27	138.0	22.0	45	152.0	16.0	−14.0	[−23.5; −4.5]	6.3%
**Random effects model**	**572**			**600**			**−22.7**	**[−28.9; −16.5]**	**100.0%**

Heterogeneity: I^2 = 84%, τ^2 = 136.1, p < 0.01

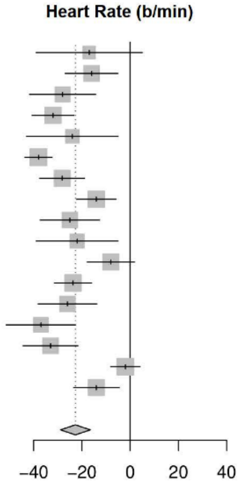

E

Study	HF Total	HF Mean	HF SD	Control Total	Control Mean	Control SD	MD	95%-CI	Weight
Bhella 2011	11	8.10	2.0	13	9.90	1.5	-1.8	[-3.2; -0.4]	20.0%
Fu 2011	48	11.00	2.9	39	18.60	1.9	-7.6	[-8.6; -6.6]	20.6%
Hearon 2019	18	10.40	2.2	18	12.70	2.9	-2.3	[-4.0; -0.6]	19.5%
Parovic 2019	17	10.80	2.2	20	10.50	2.3	0.3	[-1.2; 1.8]	19.9%
Sarma 2021	20	11.30	2.1	14	14.00	2.0	-2.7	[-4.1; -1.3]	20.0%
**Random effects model**	**114**			**104**			**-2.9**	**[-5.5; -0.3]**	**100.0%**

Heterogeneity: I^2 = 95.9%, τ^2 = 8.2722, p < 0.0001

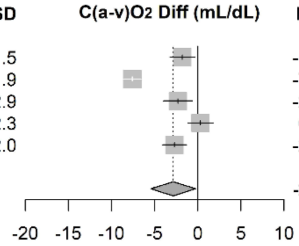

Supplement Figure 2. Systolic Blood pressure (SBP) (mmHg; Panel A) and Diastolic Blood pressure (DBP) (mmHg; Panel B) in HF and CON.

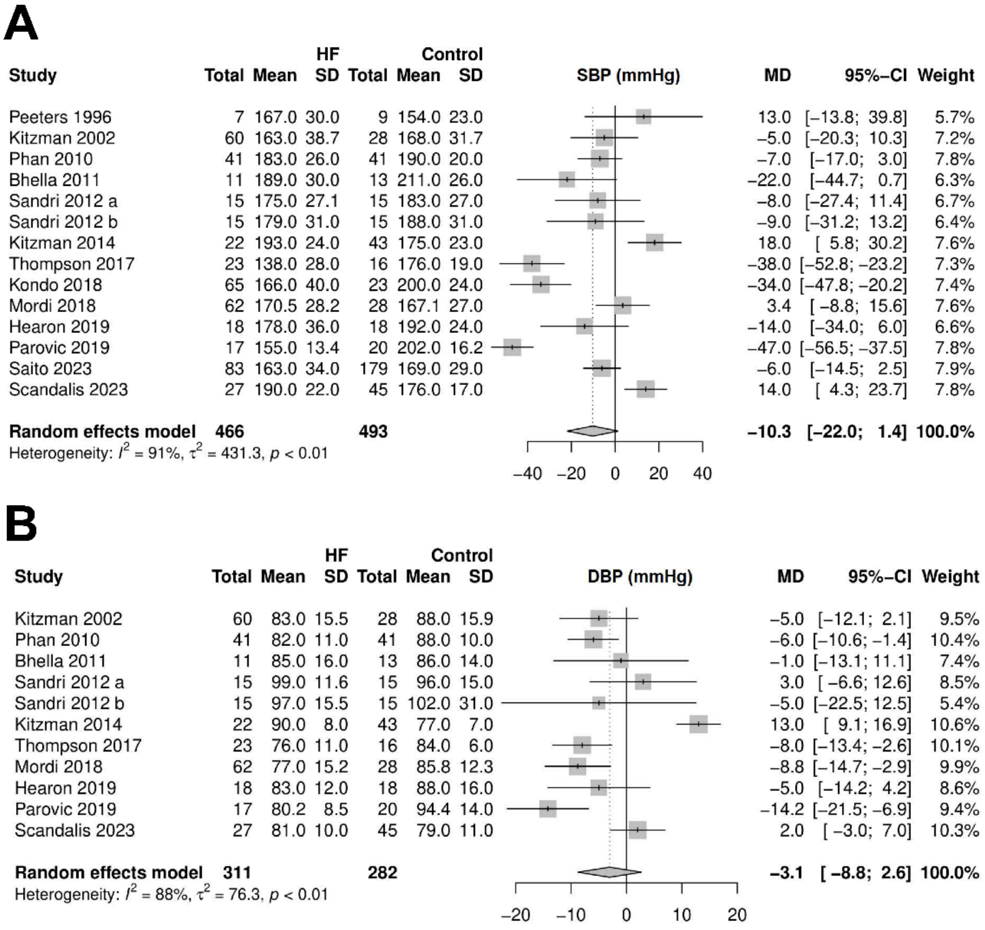

Supplement Figure 3. Funnel plots and Egger´s Test for VO2peak (mL/min/kg) in HF vs. CON (Panel A); Young-Old (YO) vs. Middle-Old (MO) HF (Panel B); MO vs. Oldest Old (OO) HF (Panel C).

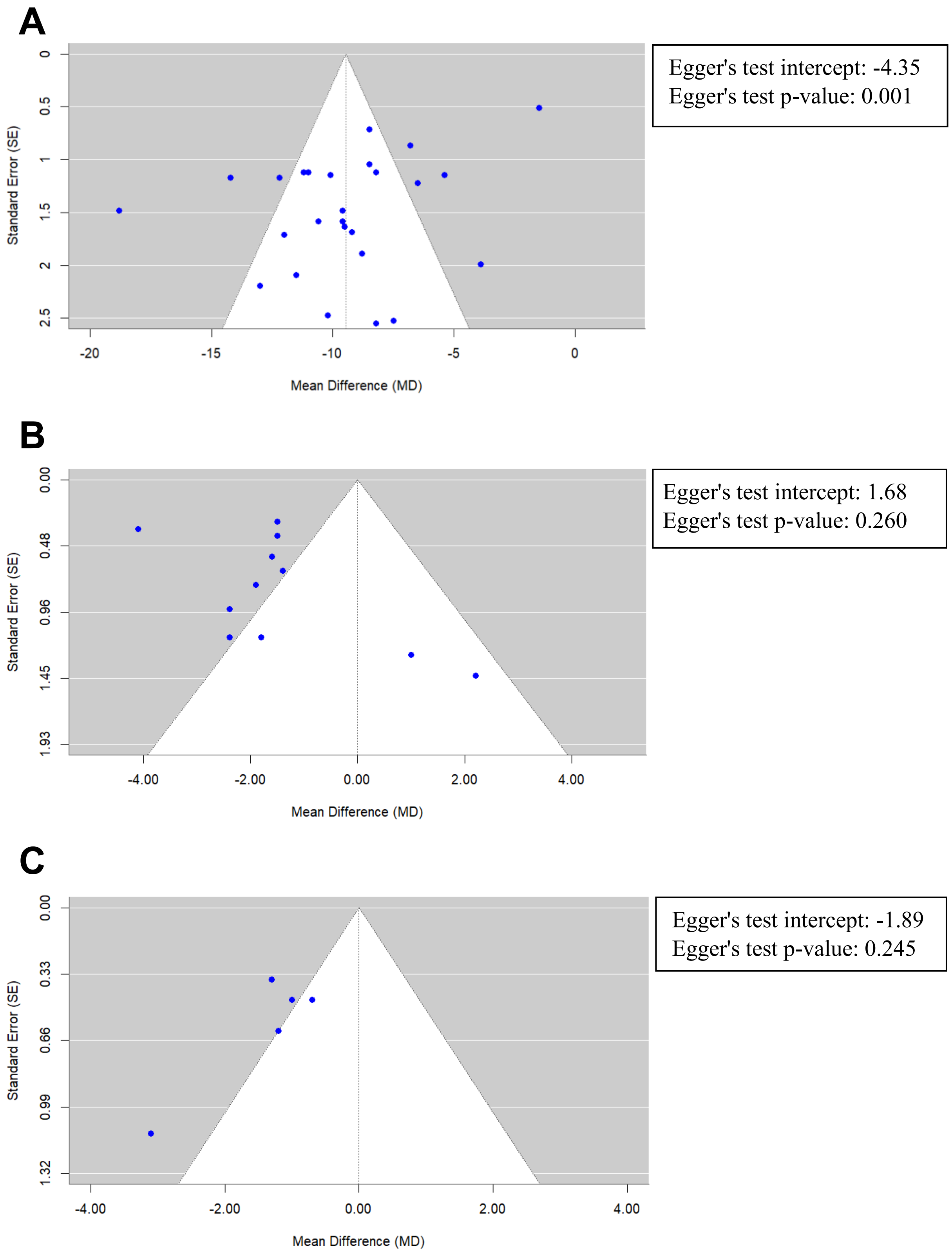

Supplement Table 1. Results of study quality assessment using the Axis Tool.

	Peeters 1996	Toth 1997	Kitzman 2002	Nishio 2003	Witte 2004	Borlaug 2006	Scardovi 2007	Williams 2007	Forman 2009	Miller 2009	Munkvik 2010	Phan 2010	Beale 2011
Were the aims/objectives of the study clear?	1	1	1	1	1	1	1	1	1	1	1	1	1
Was the study design appropriate for the stated aim(s)?	1	1	1	1	1	1	1	1	1	1	1	1	1
Was the sample size justified?	0	0	0	0	0	0	0	0	0	0	0	0	0
Was the target/reference population clearly defined? (Is it clear who the research was about?	1	1	1	1	1	1	1	1	1	1	1	1	1
Was the sample frame taken from an appropriate population base so that it closely represented the target/reference population under investigation?	1	1	1	1	0	1	1	1	1	1	0	1	1
Was the selection process likely to select subjects/participants that were representative of the target/reference population under investigation?	Unsure	1	1	1	0	1	1	1	1	1	1	0	0
Were measures undertaken to address and categorise non-responders?	0	0	0	0	0	0	0	0	0	0	0	0	0
Were the risk factor and outcome variables measured appropriate to the aims of the study?	1	1	1	1	1	1	1	1	1	1	1	1	1
Were the risk factor and outcome variables measured correctly using instruments/measurements that had been trialled, piloted or published previously?	1	1	1	1	1	1	1	1	1	1	1	1	1
Is it clear what was used to determine statistical significance and/or precision estimates? (e.g. p-values, confidence intervals)	1	1	1	1	1	1	1	1	1	1	1	1	1
Were the methods (including statisitcal methods) sufficiently described to enable them to be repeated?	1	0	1	1	1	1	1	1	0	0	1	1	1
Were the basic data adequately described?	1	0	1	1	0	1	0	0	1	0	0	1	0
Does the response rate raise concerns about non-response bias?	0	0	0	0	0	0	0	0	0	0	0	0	0
If appropriate, was information about non-responders described?	0	0	0	0	0	0	0	0	0	0	0	0	1
Were the results internally consistent?	1	1	1	1	1	1	0	1	1	1	1	1	1
Were the results presented for all the analyses described in the methods?	1	1	1	1	1	1	1	1	1	1	1	1	1
Were the authors' discussions and conclusions justified by the results?	1	1	1	1	1	1	1	1	1	1	1	1	1
Were the limitations of the study discussed?	0	0	1	0	1	1	0	1	1	0	0	1	1
Were there any funding sources or conflicts of interests that may affect the authors' interpretation of the results?	0	Unsure	1	0	Unsure	0	0	0	0	1	0	0	1
Was ethical approval or consent of participation attained?	1	0	1	1	0	1	1	1	0	1	1	1	1
	**13**	**11**	**16**	**14**	**11**	**15**	**12**	**14**	**13**	**13**	**12**	**14**	**15**

	Bhella 2011	Fu 2011	Savage 2011	Sandri 2012	Ciolac 2013	Rullman 2013	Tan 2013	Zavin 2013	Kitzman 2014	Carubelli 2015	Olson 2016	Tarperi 2016	Nanayakkara 2017
Were the aims/objectives of the study clear?	1	1	1	1	1	1	1	1	1	1	1	1	1
Was the study design appropriate for the stated aim(s)?	1	1	1	1	1	1	1	1	1	1	1	1	1
Was the sample size justified?	0	0	0	1	0	0	0	0	0	0	0	0	0
Was the target/reference population clearly defined? (Is it clear who the research was about?	1	1	1	1	1	1	1	1	1	1	1	1	1
Was the sample frame taken from an appropriate population base so that it closely represented the target/reference population under investigation?	1	1	Unsure	1	0	1	1	1	1	1	1	1	1
Was the selection process likely to select subjects/participants that were representative of the target/reference population under investigation?	1	1	Unsure	0	0	1	0	1	1	1	Unsure	0	Unsure
Were measures undertaken to address and categorise non-responders?	0	0	0	0	0	0	0	0	1	1	0	0	0
Were the risk factor and outcome variables measured appropriate to the aims of the study?	1	1	1	1	1	1	1	1	1	1	1	1	1
Were the risk factor and outcome variables measured correctly using instruments/measurements that had been trialled, piloted or published previously?	1	1	1	1	1	1	1	1	1	1	1	1	1
Is it clear what was used to determine statistical significance and/or precision estimates? (e.g. p-values, confidence intervals)	1	1	1	1	1	0	1	1	1	1	1	1	0
Were the methods (including statisitcal methods) sufficiently described to enable them to be repeated?	1	1	1	1	1	0	0	1	1	1	1	0	1
Were the basic data adequately described?	1	1	1	1	1	0	1	1	1	1	1	1	1
Does the response rate raise concerns about non-response bias?	0	0	1	0	0	0	0	0	0	0	0	0	0
If appropriate, was information about non-responders described?	0	0	1	0	0	0	0	0	0	1	0	0	0
Were the results internally consistent?	1	1	1	1	1	1	1	1	1	1	1	1	1
Were the results presented for all the analyses described in the methods?	1	1	1	1	1	1	1	1	1	1	1	1	1
Were the authors' discussions and conclusions justified by the results?	1	1	1	1	1	1	1	1	1	1	1	1	1
Were the limitations of the study discussed?	1	1	1	1	1	1	1	1	1	1	1	0	1
Were there any funding sources or conflicts of interests that may affect the authors' interpretation of the results?	0	0	0	1	1	1	1	0	0	Unsure	0	0	0
Was ethical approval or consent of participation attained?	1	1	1	1	1	1	1	1	1	0	1	1	1
	**15**	**15**	**15**	**16**	**14**	**13**	**14**	**15**	**16**	**16**	**14**	**12**	**13**

	Thompson 2017	Kato 2018	Mahmod 2018	Kondo 2018	Mordi 2018	Hearon 2019	Parovic 2019	Rullman 2020	Sarma 2021	Peterman 2021	Saito 2023	Scandalis 2023	
Were the aims/objectives of the study clear?	1	1	1	1	1	1	1	1	1	1	1	1	
Was the study design appropriate for the stated aim(s)?	1	1	1	1	1	1	1	1	1	1	1	1	
Was the sample size justified?	0	0	0	0	0	0	0	0	0	0	0	0	
Was the target/reference population clearly defined? (Is it clear who the research was about?	1	1	1	1	1	1	1	1	1	1	1	1	
Was the sample frame taken from an appropriate population base so that it closely represented the target/reference population under investigation?	1	1	1	1	1	1	0	1	1	1	1	1	
Was the selection process likely to select subjects/participants that were representative of the target/reference population under investigation?	1	1	1	1	1	1	0	1	1	1	1	1	
Were measures undertaken to address and categorise non-responders?	0	0	0	1	1	0	0	0	0	0	1	0	
Were the risk factor and outcome variables measured appropriate to the aims of the study?	1	1	1	1	1	1	1	1	1	1	1	1	
Were the risk factor and outcome variables measured correctly using instruments/measurements that had been trialled, piloted or published previously?	1	1	1	1	1	1	1	1	1	1	1	1	
Is it clear what was used to determine statistical significance and/or precision estimates? (e.g. p-values, confidence intervals)	0	1	1	1	1	1	1	1	1	1	1	1	
Were the methods (including statisitcal methods) sufficiently described to enable them to be repeated?	0	1	1	1	0	1	0	0	1	1	1	1	
Were the basic data adequately described?	1	1	1	1	1	1	1	1	1	1	1	1	
Does the response rate raise concerns about non-response bias?	0	0	0	0	0	0	0	0	0	0	0	0	
If appropriate, was information about non-responders described?	0	0	0	0	0	0	0	0	0	0	1	0	
Were the results internally consistent?	1	1	1	1	1	1	1	1	1	1	1	1	
Were the results presented for all the analyses described in the methods?	1	1	1	1	1	1	1	0	1	1	1	1	
Were the authors' discussions and conclusions justified by the results?	1	1	1	1	1	1	1	1	1	1	1	1	
Were the limitations of the study discussed?	0	1	1	1	1	1	1	1	1	1	1	1	
Were there any funding sources or conflicts of interests that may affect the authors' interpretation of the results?	0	1	1	0	Unsure	0	1	0	0	1	1	1	
Was ethical approval or consent of participation attained?	1	0	1	1	1	1	1	1	1	1	1	1	**Total**
	**12**	**15**	**16**	**16**	**15**	**15**	**13**	**13**	**15**	**16**	**18**	**16**	**14.3**
